# Comparative transcriptional analysis identifies genes associated with the attenuation of *Theileria parva* infected cells after long-term in vitro culture

**DOI:** 10.1038/s41598-024-59197-y

**Published:** 2024-04-18

**Authors:** Elisabeth M. D. L. van der Heijden, Lucas Lefevre, Anton Gossner, Thomas Tzelos, Timothy K. Connelley, Musa A. Hassan

**Affiliations:** 1 Division of Infectious Diseases and Immunology, Department of Biomolecular Health Sciences, Faculty of Veterinary Medicine, Utrecht University, Utrecht, The Netherlands; 2 Division of Immunology, The Roslin Institute, University of Edinburgh, Edinburgh, UK; 3 Centre for Tropical Livestock Genetics and Health, Easter Bush Campus, Edinburgh, UK; 4 Present Address: Moredun Research Institute, Pentlands Science Park, Bush Loan, Penicuik, EH26 0PZ UK

**Keywords:** Transcriptomics, Gene expression, Sequencing

## Abstract

Autologous administration of attenuated *Theileria parva*-infected cells induces immunity to *T. parva* in cattle. The mechanism of attenuation, however, is largely unknown. Here, we used RNA sequencing of pathogenic and attenuated *T. parva*-infected T-cells to elucidate the transcriptional changes underpinning attenuation. We observed differential expression of several host genes, including *TRAIL*, *PD-1*, *TGF-β* and granzymes that are known to regulate inflammation and proliferation of infected cells. Importantly, many genes linked with the attenuation of the related *T. annulata*-infected cells were not dysregulated in this study. Furthermore, known *T. parva* antigens were not dysregulated in attenuated relative to pathogenic cells, indicating that attenuation is not due to enhanced immunogenicity. Overall this study suggests that attenuation is driven by a decrease in proliferation and restoration of the inflammatory profile of *T. parva*-infected cells. Additionally, it provides a foundation for future mechanistic studies of the attenuation phenotype in *Theileria*-infected cells.

## Introduction

*Theileria parva* (*T. parva*) is a tick-transmitted obligate intracellular hemoprotozoan parasite in the order of the Piroplasmidae^1^. Infection with *T. parva* causes East Coast Fever (ECF), a devastating disease of high morbidity and mortality, in cattle in sub-Saharan Africa^2^. The life-cycle of *T. parva* is complex, relying on the bovine host and a tick vector, *Rhipicephalus appendiculatus* (*R. appendiculatus)*^3^. Infective *T. parva* sporozoites injected into the host by blood-feeding ticks rapidly invade host lymphocytes—predominantly T- and B-cells^4,5^—and undergo schizogonous division to become multinucleate schizonts. This process is associated with a cancer-like transformation and uncontrolled proliferation of schizont-infected lymphocytes^6^, which give rise to the classical symptoms associated with ECF, such as generalized lymphadenopathy, respiratory distress, fever and anorexia^7^. The schizont-induced transformation effectively immortalizes the cell, and association of the parasite with the mitotic spindle in the cytosol of the host cell ensures concomitant infection of daughter cells with each division^8^. Unlike the transformation seen, for instance in cancerous cells^4^, *Theileria*-induced transformation is reversible by compounds such as buparvaquone.

Given the proliferative phenotype of *T. parva*-infected cells, they are readily maintained in in vitro culture by serial passaging. Autologous *T. parva*-infected T-cell-lines (TpM) have been shown to be highly pathogenic when administered to naïve animals early after establishment in vitro (within ~ 7 days), leading to the development of severe clinical symptoms of ECF, and death of the animal if left untreated^9,10^. However, after long-term culture (≥ 42 days), in vitro established autologous TpM exhibit an attenuated phenotype, inducing only mild and transient clinical signs in naïve animals which subsequently recover and gain immunity to re-infection^9^. As a result, autologous TpM can be used as an alternative strategy to the current infection-and-treatment vaccination method^11,12^. However, the molecular processes through which the attenuated phenotype is achieved are largely unknown. Furthermore, it is currently not known whether this phenomenon is driven by host- or parasite-derived factors. Elucidating the mechanisms underlying TpM attenuation would further our understanding of the host–pathogen interactions and help identify potential novel targets for vaccine development and therapeutics for ECF.

Although a significant amount of work studying the attenuation of *Theileria annulata*^13–15^ has been completed, very little equivalent work has been done for *T. parva*. The goal of this study, therefore, was to characterize the transcriptomic changes accompanying *T. parva*-infected bovine T-cells transitioning from pathogenic to attenuated phenotypes. To this end, the transcriptomes of naïve CD4^+^ T-cells (control) and in vitro-derived TpMs cultured for 7 and 69 days post-infection were obtained using high-throughput RNA sequencing and analysed. We found that in both day 7 (pathogenic) and day 69 (attenuated) TpM, there was up- and downregulation of cancer- and immunity-related host genes, respectively compared to naïve CD4^+^ T-cells. However, direct comparison of the day 69 cells to day 7 cells revealed three major changes associated with attenuation; a relative reactivation of a pro-inflammatory phenotype, curtailment of proliferation and a shift in metabolism.

## Materials and methods

### Ethics statement

All animal experiments were completed under a license from the UK government granted under the UK Animals (Scientific Procedures) Act 1986. All animal experiments were reviewed and approved by The Roslin Institute Animal Welfare and Ethics Review Board, and carried out according to relevant guidelines and regulations. All methods are reported in accordance with ARRIVE guidelines.

### Study animals

Six 4–6 months old healthy Holstein–Friesian (HF) cattle housed at the University of Edinburgh Dryden Farm were used in this study. All animals were bled for the generation of *T. parva* infected cell-lines as described below. Animals were euthanized by a Schedule 1 approach in accordance with Home Office (UK government regulations). The agent used was pentobarbital sodium, administered intra-venously.

### Isolation of CD4^+^ T-cells

Blood was collected by jugular venepuncture and peripheral blood mononuclear cells (PBMC) were isolated using standard density gradient centrifugation. PBMC were suspended in standard culture medium (SCM) consisting of RPMI-1640 (Thermo Fisher Scientific, Cheshire, UK), supplemented with 10% FBS, 50mM 2-mercaptoethanol (Merck, Gillingham, UK) and 1X penicillin–streptomycin-glutamine (Thermo Fisher Scientific), and MACS sorted using the CD4 specific antibodies listed in Table 1. Briefly, PBMC were stained with 0.5µg/mL of ILA-12 antibody at 4˚C for 15 min and washed in SCM by centrifugation at 4–8˚C 300 × *g* for 5 min. Cells were then resuspended in SCM and incubated with anti-IgG beads at 4˚C for 10 min, washed as above, and run through an LS-column (Miltenyi Biotec, Surrey, UK) to enrich for CD4^+^ cells. A minimum purity of 95%, checked as described below, was achieved for each sample.

**Table 1:** Antibodies and stains used for MACS sorting, immunofluorescence microscopy and flowcytometry.

Antibody/Stain	Specificity	Isotype	Source
*MACS sorting*			
ILA-12	CD4	IgG2a	In-house
*Immunofluorescence microscopy*			
ILS-40	PIM (*T. parva*)	IgG2a	In-house
IgGAM-FITC	Mouse IgG, IgA & IgM	Goat polyclonal	Merck
DAPI	Nuclei	n/a	Thermofisher Scientific
*Flowcytometry*			
CC8-PE	CD4	IgG2a	Thermofisher Scientific

### *Theileria parva* infected CD4^+^ T-cell-lines

*Theileria parva* infected cell-lines (TpM) were established in vitro by exposing 2 × 10^7^ CD4^+^ T-cells in 1mL of SCM to saturating levels of fresh *T. parva* Muguga sporozoites, as previously described^16^. At day 7 post-infection, cell aliquots from two TpM lines were separately harvested, sequentially washed once in SCM and PBS, with centrifugation after each wash at 400 × *g* for 5 min. Cell viability was assessed using Trypan Blue (Thermo Fisher Scientific) and cells resuspended in PBS at 2 × 10^4^/mL. Two animals were inoculated with 5mL of autologous TpM cell-lines in the left side of the neck, +− 10–15 cm dorsoanterior to the prescapular lymph node (PSLN). Animals were closely monitored for the development of pathology and euthanized within 1 week post-infection due to severe disease symptoms, which confirmed pathogenicity of the day 7 TpM cell-lines. The rest of the cell-lines were cultured in 75 cm^2^ culture flasks by serial passaging (1:4) every 2–3 days until day 69.

### Confirmation of host cell infection and phenotype

Confirmation of infection of each TpM cell-line was performed by indirect immunofluorescent staining using the antibodies in Table 1 and visualized under a microscope. Briefly, 5 × 10^4^ cells were loaded onto slides by cytospin centrifugation and fixed in acetone before staining with 1µg/mL ILS-40 primary antibody (specific for polymorphic immunodominant molecule (PIM)) and subsequently an anti-murine IgGAM-FITC secondary antibody, 4',6-diamidino-2-phenylindole (DAPI) counterstain. Slides were washed with PBS twice in between stains and rinsed in dH_2_O before mounting and visualisation under a fluorescent microscope. The infection rate was determined as the percentage of infected cells (PIM^+^ cells × 100%/DAPI^+^ host cell nuclei).

In order to confirm that TpM cell-lines consisted of CD4^+^ T-cells, phenotyping was performed with flow cytometry using the antibodies in Table 1. Briefly, an aliquot of each cell-line was harvested during passage, washed, and brought to 1 × 10^6^ cells/mL in FACS buffer (PBS containing 0.5% BSA). 100 µL of cell suspension was stained with 10µL of PE-conjugated CC8 antibody at 4 °C for 15 min. The cells were washed with FACS buffer twice by pulse centrifuging to 600 × *g* at 4 °C and resuspended in FACS buffer. Data was acquired on the FACS Aria IIIu (BD Biosciences).

### Characterization of the transcriptome of TpM cell-lines

At days 7 and 69 post-infection, total RNA was extracted from 3 × 10^6^ cells of each TpM line using the miRNeasy mini kit (Qiagen, Manchester, UK) according to the manufacturer’s protocol, with an additional DNAse treatment step using the TURBO DNA-free kit (Ambion, Banchory, UK). RNA was also extracted from 3 × 10^6^ uninfected CD4^+^ T-cells (day 0, controls) using the same protocols. The quantity and integrity of the resulting total RNA samples were analysed using the Qubit BR RNA assay (Thermo Fisher Scientific) and high sensitivity RNA screentape on the TapeStation (Agilent, Manchester, UK), respectively, according to the manufacturers’ protocols.

Library preparation and sequencing was carried out at the Wellcome Trust Clinical Research facility at the University of Edinburgh (Scotland, UK). Briefly, the libraries were separately prepared from 500 ng of each total-RNA sample using the NEBNEXT Ultra II Directional RNA Library Prep kit (New England BioLabs (NEB), Hitchin, UK #7760) and the Poly-A mRNA magnetic isolation module (NEB #E7490) according to the provided protocol. Following purification, the mRNA was fragmented using divalent cations under elevated temperature, primed with random hexamers and reverse transcribed into first strand cDNA. RNA templates were removed and a replacement strand synthesised incorporating dUTP in place of dTTP to generate ds cDNA. AMPure XP beads (Beckman Coulter, High Wycombe, UK #A63881) were then used to separate the ds cDNA from the second strand reaction mix, providing blunt-ended cDNA. Multiple indexing adapters were then ligated to the ends of the ds cDNA to prepare them for hybridisation onto a flow cell, before 11 cycles of PCR were used to selectively enrich those DNA fragments that had adapter molecules on both ends and amplify the amount of DNA in the library suitable for sequencing. After amplification libraries were purified using AMPure XP beads. Libraries were quantified by fluorometry using the Qubit dsDNA HS assay and assessed for quality and fragment size using the Agilent Bioanalyser with the DNA HS Kit (Agilent, #5067-4626). Libraries were multiplexed in an equimolar pool based on Qubit and Bioanalyser assay results and spiked with PhiX Control v3 (Illumina Inc, Cambridge, UK, #FC-110-3001). Sequencing was performed on the NextSeq 2000 platform (Illumina Inc, #20038897) using NextSeq 2000 P3 Reagents (100 Cycles) (Illumina Inc, #20040559).

### Bioinformatic analysis pipeline

Raw reads were subjected to quality control using FastQC (v 0.11.9)^17^ and subsequently trimmed of Illumina adapters and poor quality reads (using the default cut-off Phred score of 20) in TrimGalore (v 0.6.4)^18^, with the following parameters: --illumina --length 35 --paired. Next, the reads were pseudoaligned to Ensembl release 108^19^ bosTau9 (ARS-UCD1.2)^20^ and PiroplasmDB release 60^21^ *T. parva* Muguga (GCA_000165365.1, Jun 11, 2020)^22^ transcripts respectively, using kallisto (v 0.46.1)^23^ with default parameters and 10 bootstraps. Transcript-level abundances were imported into R^24^ and summarized into gene-level counts using tximport (v 1.24.0)^25^. Differential expression analysis was carried out using DESeq2 (v 1.36.0)^26^ with log-fold shrinkage (apeglm^27^). The Database for Annotation, Visualization and Integrated Discovery (DAVID)^28^ was used to explore enriched gene ontology terms and clustering of KEGG^29–31^ pathways based on the lists of differentially expressed bovine genes. Next, gene set enrichment analysis (GSEA) was performed using GSEA-P (v 4.3.2)^32,33^ with "Hallmark” gene sets^34^ from the Molecular Signature Database^35^ on the Log2 fold change (L2FC) pre-ranked *B. taurus* differentially expressed gene lists. Finally, leading edge (LE) analysis was performed on the significantly enriched gene sets to determine which genes contributed most to the enrichment of (multiple) gene sets. The R packages ggplot2^36^ and ComplexHeatmap^37^, and Graphpad Prism v9.3.1 and BioRender.com were used for visualization of the results.

### Statistics and reproducibility

The level of statistical significance was set at false discovery rate (FDR) ≤ 0.05 and L2FC ≥ 2 as determined by DESeq2, or FDR ≤ 0.05 as determined by GSEA-P.

## Results and discussion

### Establishing a system to capture the transcriptomic profiles of bovine CD4^+^ T-cells during pathogenic and attenuated phases of *Theileria parva* infection in vitro

*T. parva*-transformed CD4^+^ T-cell-lines (TpM) from 6 healthy HF calves were established ex vivo by infection of purified CD4^+^ T-cells derived from PBMC with saturating levels of *T. parva* (Muguga strain) and maintained in vitro by passaging every 2–3 days for up to 69 days post-infection. To capture the changes in the host and parasite transcriptomes that occur in the early and late stages of *T. parva* infection, high throughput RNA sequencing (RNA-seq) was performed on uninfected CD4^+^ T-cells (day 0—controls), and TpM aliquots taken on day 7 (pathogenic) and day 69 (attenuated) post-infection. Two of the six TpM lines could not be maintained in culture for the intended 69-day period. Flow cytometry and immunohistochemistry analyses performed at each time-point confirmed that ≥ 95% of the cells were CD4^+^ T-cells and that on days 7 and 69, ≥ 95% of the cells were infected with *T. parva* (i.e. contained *T. parva* schizonts). To verify that day 7 cultured TpM were pathogenic, 1 × 10^5^ cells were administered to the autologous naïve animals, who subsequently developed acute and severe classical symptoms of ECF^38^ and were euthanized within 1 week of infection (n = 2, data not shown). From each sample, the number of reads that passed quality-control was between 31 M and 46 M, and the percentage of reads mapping uniquely to the bovine reference genome was between 70 and 87%. One TpM collected on day 69 was excluded because the RNA-seq reads showed poor alignment to both the host and parasite transcriptomes. The final dataset thus, comprised 6 (n = 6), 6 (n = 6), and 3 (n = 3) biological replicates at day 0, 7 and 69 post-infection, respectively.

### Transcriptional changes in bovine CD4^+^ T-cells induced by infection with *T. parva*

**Figure 1:**
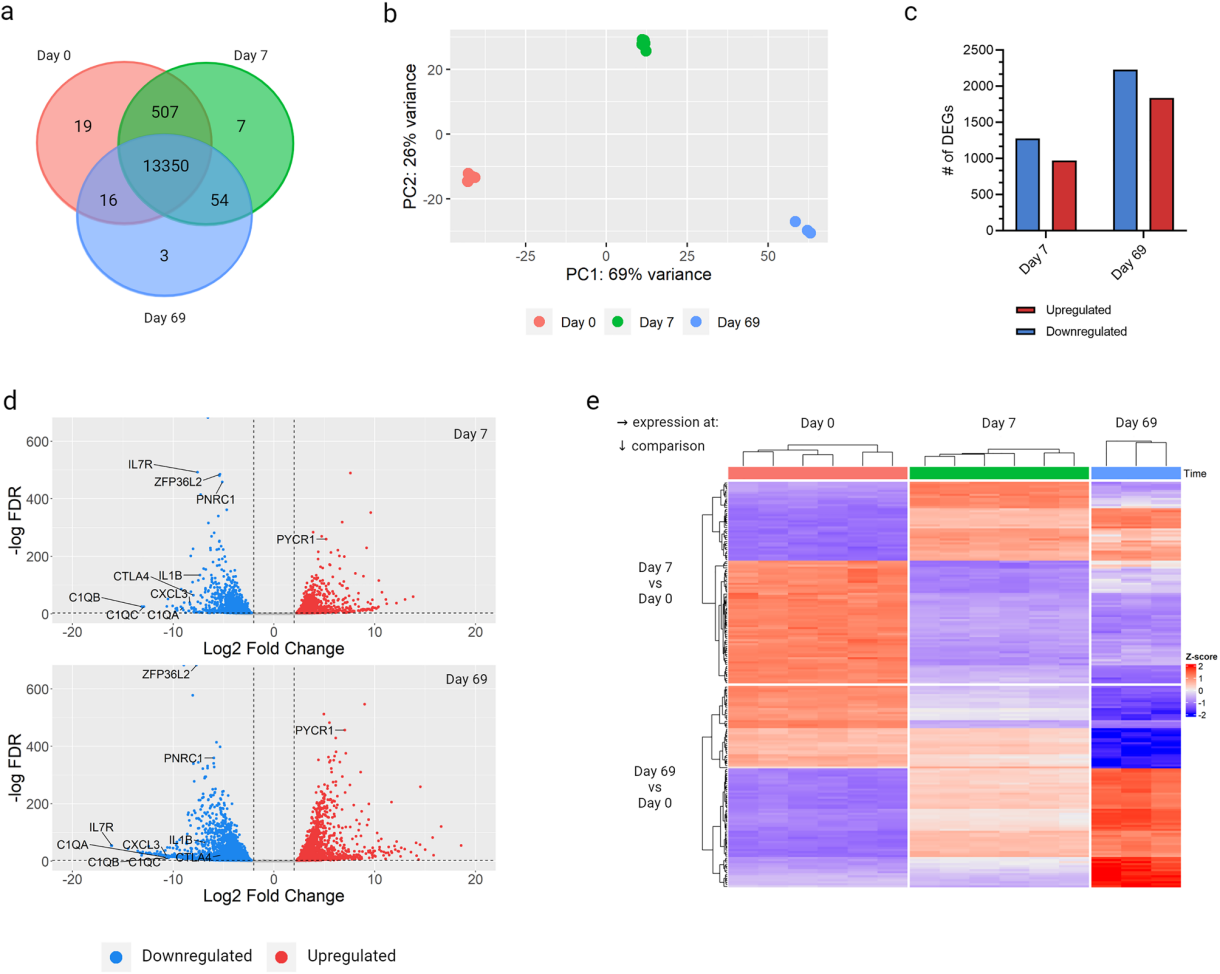
Expression of *Bos taurus* genes in different states of *Theileria parva* infection of bovine CD4^+^ T-cells. (**a**) The total number of expressed genes at day 0 (red), day 7 (green) and day 69 (blue). (**b**) Principal component analysis plot showing the first and second principle components; based on variance stabilized transformed counts of all expressed genes. (**c**) The number of significantly upregulated (red) or downregulated (blue) genes at day 7 and day 69, relative to day 0, as determined by DE analysis using DESeq2 (FDR ≤ 0.05 and L2FC |≤ 2|). (**d**) Distribution of all significantly DEGs according to the L2FC and −log(adjusted *p*-value) at day 7 and day 69, relative to day 0. (**e**) Expression of the top 100 significantly DEGs at day 7 and day 69, relative to day 0, displayed as z-score transformed counts. Created with ComplexHeatmap v2.12.1 and ggplot2 v3.4.2 in R Studio v2023.03.1, Graphpad Prism v9.3.1 and BioRender.com.

To study the transcriptional changes induced in T-cells following infection with *T. parva*, differential gene expression analyses of day 7 and 69 TpMs, relative to uninfected (day 0) controls was performed. Downstream analysis was restricted to 13,956 bovine genes that had at least 10 uniquely mapped RNA-seq reads in at least three biological replicates (the minimum number of biological replicates at each time-point). Most genes (13,350, 95.6%) were expressed at all time-points, with only 19, 7 and 3 genes uniquely expressed at day 0, 7 and 69, respectively, and 577 genes (4.1%) expressed at 2 out of the 3 time-points (Fig. 1a). Exploratory principal component analysis (PCA) showed that infection status, captured by PC1, was the major source of transcriptional heterogeneity among the samples (Fig. 1b).

In total, 5,713 unique genes were differentially expressed (log2fold change ≥ 2 and FDR ≤ 0.05) in at least one time-point relative to the controls (Supplementary File 1). Despite the distinct and well-characterised phenotypic changes evident in TpM (i.e. transformation), compared to uninfected CD4^+^ T-cells, a majority of genes (~ 70%) were not significantly dysregulated at either day 7 or day 69. In comparison to day 0 uninfected cells, a larger proportion of genes were downregulated than upregulated in TpM, with 1275 and 2230 genes down-regulated, and 970 and 1837 genes up-regulated at day 7 and 69, respectively (Fig. 1c). This suggests infection with *T. parva* predominantly was associated with an inhibition rather than activation of specific host gene expression and biological processes.

#### Day 7 vs. Day 0

Preliminary observation of the most differentially expressed genes at day 7 revealed that 31 out of the 100 most downregulated DEGs, including *C1QA-C*, *IL1B*, *IL7R*, *CTLA4* and *CXCL3*, were associated with immune and inflammatory responses (Fig. 1d and Supplementary File 1), suggesting that early in infection the parasite induces an anti-inflammatory profile. Subversion of the local inflammatory milieu during the early phase of infection may contribute to the dysregulation of the primary host T-cell response that has been reported previously^39^. This in vivo dysregulation observed in primary *T. parva* infection results in the generation of a non-protective and aberrant T-cell response, which has been proposed to facilitate the parasite reaching the critical mass required for its survival and subsequent pathogenesis. Conversely, the top 10 most upregulated genes at day 7 included a number of genes associated with cell proliferation and cancer, including *FKBP10*^40^, *STC2*^41^ and *AK4*^42^. Of note, some of the genes associated with the TGF-β pathway—known to be dysregulated in leukocytes transformed by the related parasite *T. annulata*^13^—such as matrix metalloproteinase 9 (*MMP9*)^14,15^, *MMP2*, *SMAD1* and *GDF10*^43,44^, were also amongst the 100 most upregulated genes. However, the up-/down-regulation of other genes identified in a recent study of the transcriptome of *T. annulata*-infected cells (TaA)^14^ was not reproduced in our data (e.g. *IL21R*, *A2M* and *IGSF9B*; Supplementary File 1), suggesting there are both similarities and differences in the host genes that are manipulated by *T. parva* and *T. annulata* parasites to effect transformation of their host cells.

#### Day 69 vs. Day 0

Relative to day 0, more genes were differentially expressed at day 69 than at day 7; 4,067 (29%) vs 2,245 (16%), respectively (Fig. 1c)—indicating that attenuation was not predominantly associated with a reversion of the transcriptome towards a naïve CD4^+^ T-cell phenotype (Fig. 1c). However, the majority (± 83%) of day 7 top 100 differentially expressed genes (DEGs) are also differentially expressed at day 69 (Fig. 1e; top panel—comparing the central and right panes), and the majority (± 65%) of the top 100 DEGs at day 69 exhibit the same ‘direction’ of differential expression at day 7 (Fig. 1e; bottom panel—comparing the central and right panes). This suggests that there is a ‘core’ of transcriptional changes observed in TpM that are shared at day 7 and day 69 when compared to uninfected cells. As a consequence, similar to day 7, many top 100 DEG at day 69 were associated with either immunity (16/100) and cancer-like phenotypes (27/100), or both. For example, *ZFP36L2*, a known suppressor of regulatory T-cells (T_regs_)^45^, and *PNRC1*, a putative tumor suppressor^46^, were downregulated at day 69 whilst *PYCR1*, which has been shown to be important in sustaining tumors^47^ was upregulated. Of note, the majority of the genes cited as examples above showed similar up/down-regulation at both day 7 and day 69 (Fig. 1 and Supplementary File 1), reflecting the broad similarities in the transcriptional changes observed at these two time-points. However, there were also clear subsets of genes that showed disparate patterns of regulation at day 7 and 69 when compared to day 0; indicating that although there were similarities in the transcriptomic profiles of the pathogenic (day 7) and attenuated (day 69) cells, there were also differences (Fig. 1e; top and bottom panels—comparing the central and right panes).

**Figure 2:**
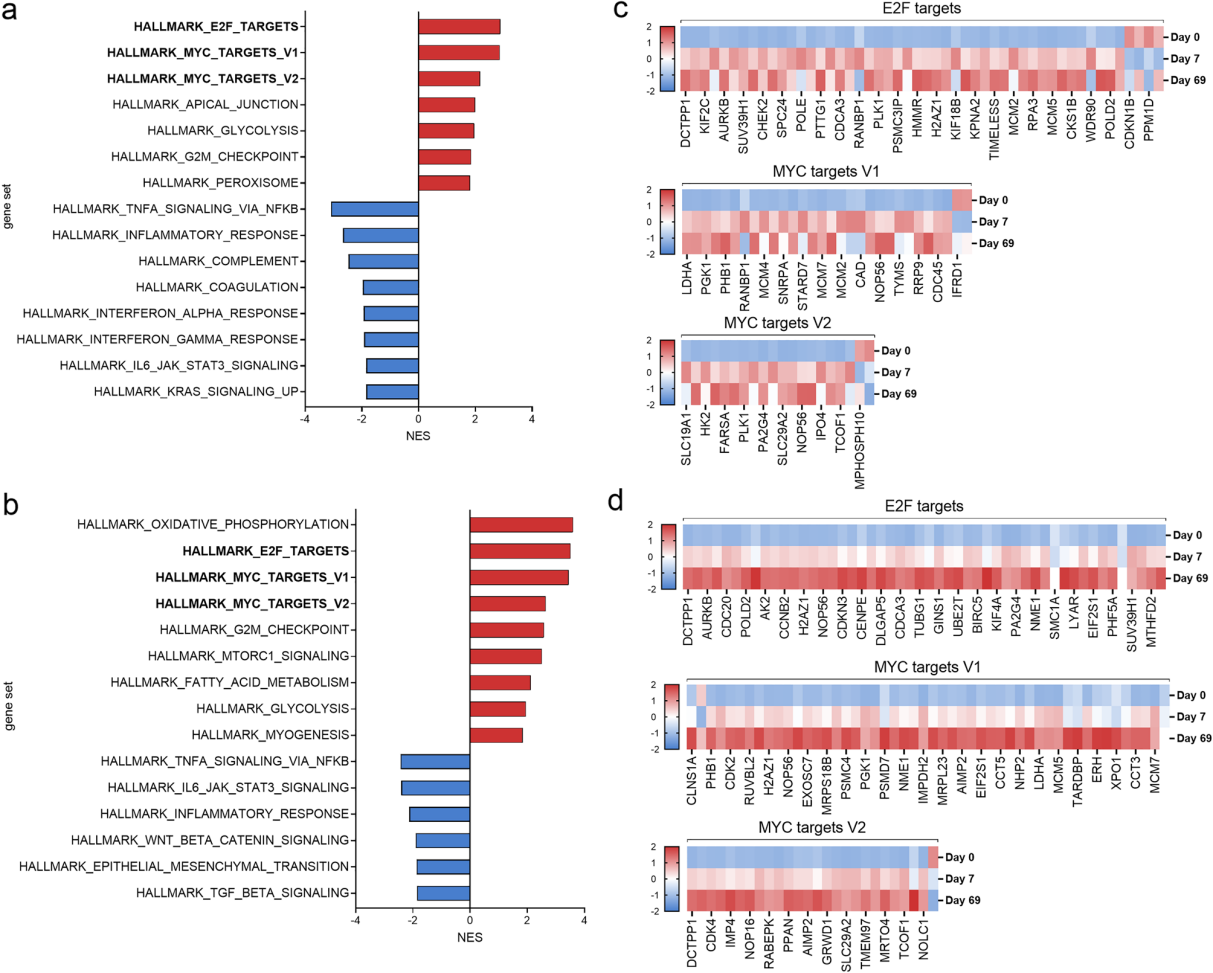
Gene set enrichment analysis of differentially expressed genes in pathogenic and attenuated cells. Normalized enrichment scores of gene sets that were either positively (red) or negatively (blue) significantly enriched at (**a**) day 7, or (**b**) day 69, relative to day 0. Expression of genes in selected significantly enriched gene sets at (**c**) day 7, or (**d**) day 69, relative to day 0. Expression is shown in z-scores of variance transformed counts. Only the top 50 genes of E2F targets at day 7 and day 69 and MYC targets V1 at day 69 are shown. Created with Graphpad Prism v.9.3.1.

To provide a more holistic contextualisation of the transcriptional differences seen at day 7 and day 69, relative to day 0, functional enrichment analyses using DAVID^28^ and GSEA^32,33^ was performed. At FDR ≤ 0.05, several gene sets, including E2F targets, MYC targets (V1 and V2), glycolysis, and G2M checkpoint, as well as gene sets related to metabolic events were positively enriched at both day 7 and day 69 (Fig. 2, Supplementary File 2, and Supplementary Figs. 1 and 2). These results are consistent with previous studies which have demonstrated that manipulation of pathways such as E2F signalling and c-Myc activation by *Theileria* are critical to the transformation of *Theileria-*infected cells^48,49^. E2F transcription factors are crucial in cell-cycle progression and an elevation in expression of these factors has been linked to malignancy and hyper-proliferation^50,51^; subversion of this pathway by *T. parva* has been demonstrated to stimulate cell-proliferation of the transformed cells^49^. The c-*myc* gene is a well-studied proto-onco gene associated with a wide variety of malignancies, and is associated with enhanced proliferation^52^; *Theileria*-transformed cells have been shown to be critically dependent on c-Myc for survival through inhibition of caspase-dependent apoptosis^48^. Enrichment of these gene sets at both day 7 and 69, and downregulation of GO terms including negative regulation of cell proliferation and growth (data not shown) provide the transcriptomic basis for the strongly proliferative phenotype that both pathogenic and attenuated TpM exhibit. Another gene set that is enriched in both pathogenic and attenuated cells is the glycolysis pathway—presumably reflecting the heightened metabolic demands associated with rapidly proliferating cells. Conversely, several gene sets associated with the host immune response, such as TNFα signalling via NF-κB, inflammatory response, and IL-6/JAK-STAT3 signalling, had a negative enrichment score at both day 7 and day 69 (Fig. 2 and Supplementary File 2), showing there are elements of the anti-inflammatory profile consistent between pathogenic and attenuated cells.

However, there were several gene sets that showed different regulation at day 7 and day 69. Notable amongst these was TGF-β signalling which was negatively enriched at day 69, but not day 7. Previous work in *T. annulata* has shown that TGF-β expression is inversely correlated with pathogenicity of transformed cells^13^ and that TGF-β signalling involves a number of other factors that are also associated with the pathogenicity of TaA (e.g. *Grb2*^53^—which in our data also is downregulated at day 69 but not day 7—Supplementary File 1), suggesting that this mechanism may contribute to the attenuation of both *T. parva* and *T. annulata*-infected cells.

Overall, the functional enrichment analyses show that at days 7 and 69, the transcriptomic profile of *T. parva*-infected cells have many shared features including those associated with anti-inflammation and enhanced proliferation (with a commensurate increase in metabolic activity). The transcriptomic profiles are consistent with the known phenotypes of *T. parva*-transformed cells (i.e. rapid proliferation and immunomodulation) and demonstrates the general validity of utilising RNA-sequencing to analyse the transcriptional profiles underlying the phenotype of *T. parva*-induced T-cell transformation. Despite the commonality in the transcriptomes of pathogenic and attenuated *T. parva* cells there were obvious differences—to further examine this the transcriptomes of the day 7 and day 69 cells were compared directly.

### Attenuation is associated with a mitigation of the anti-inflammatory profile, curtailment of proliferation and a metabolic shift

**Figure 3:**
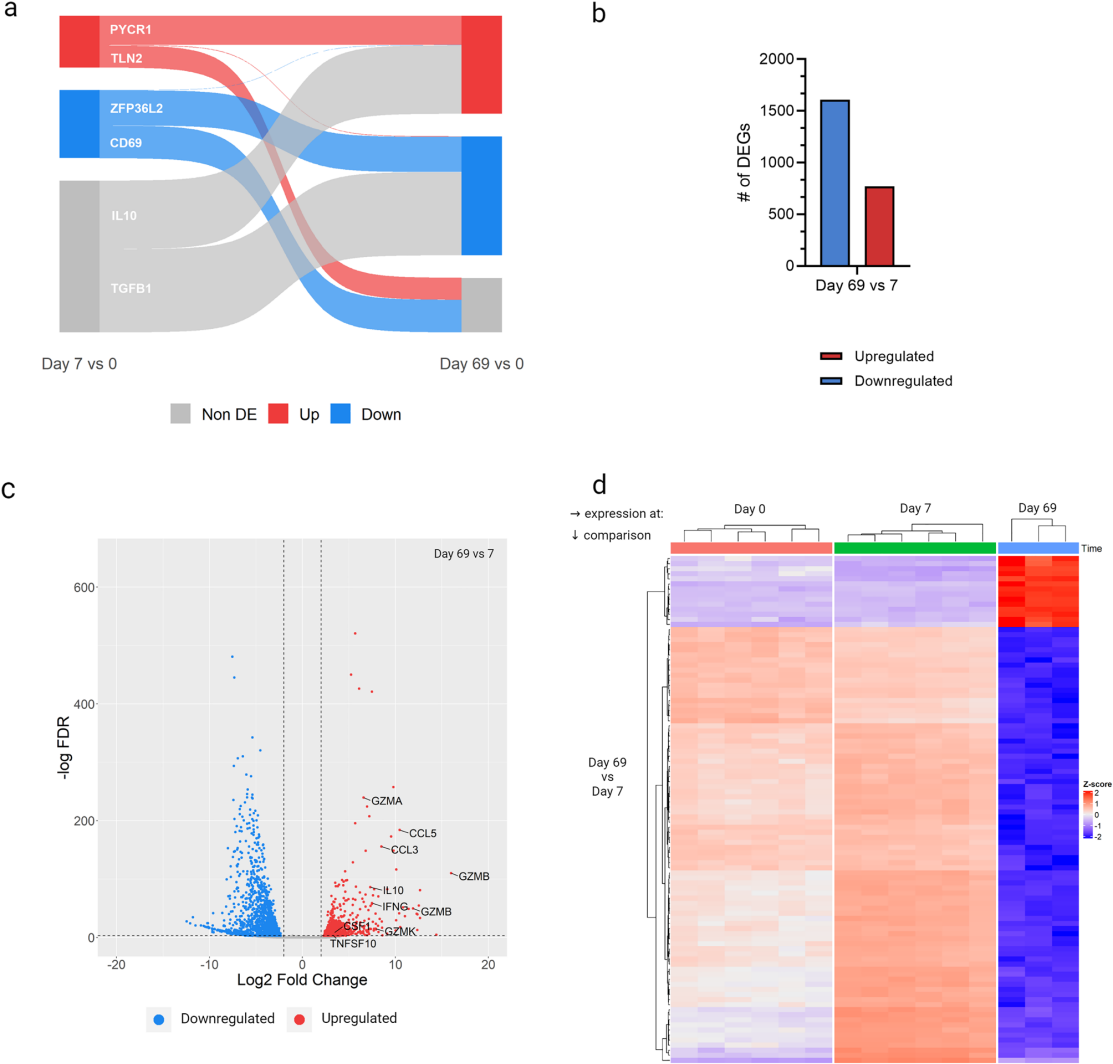
Direct comparison of attenuated to pathogenic *T. parva* infected cells. (**a**) Changes in directionality of genes significantly differentially expressed at either day 7 or day 69, relative to day 0, as determined by DE analysis using DESeq2 (FDR ≤ 0.05 and L2FC |≤ 2|). (**b**) The number of significantly upregulated (red) or downregulated (blue) genes at day 69 compared to day 7. (**c**) Distribution of all significantly DEGs according to the L2FC and −log(adjusted *p*-value) at day 69 compared to day 7. (**d**) Expression of the top 100 significantly DEGs at day 69 compared to day 7, displayed as z-score transformed counts. Created with ggsankey v.0.0.9999, ComplexHeatmap v2.12.1 and ggplot v3.4.2 in R Studio v2023.03.1 and BioRender.com. Blue = downregulated; red = up-regulated; grey = non differentially expressed (DE).

To track the changes in gene regulation as the cells transitioned from naïve CD4^+^ T-cells to pathogenic and then attenuated TpM, a Sankey plot showing genes up-, down- and non-differentially regulated at day 7 and day 69 was generated (Fig. 3a). The plot highlighted that the majority of genes that were differentially expressed at day 7 (n = 2245), remained regulated in the same direction at day 69 (n = 1208, 54%) whilst only a negligible proportion of genes (n = 15, 0.01%) inverted direction, indicating a substantial continuity in the transcriptomics of pathogenic and attenuated cells. While a high number of up-/down-regulated genes at day 7 become non-differentially regulated at day 69 (n = 1022, 46%), a greater number of genes (n = 2844) that were non-differentially regulated at day 7 became up-/down-regulated at day 69 re-iterating that attenuation is associated with a greater perturbation of the transcriptome than pathogenicity and that may be associated with disruption of more cellular pathways and processes.

In direct day 69 vs. day 7 comparison, in total 2378 genes were differentially expressed; of which 770 and 1608 were up- and down-regulated, respectively, in attenuated (day 69), compared to pathogenic (day 7) cells (Fig. 3b). Amongst the genes most upregulated in day 69 cells were a suite of genes associated with immune/inflammatory responses, including a number of chemokine ligands (*CCL1*, *CCL3* and *CCL5*), interferon-γ (*IFNG*), *IL-10*, and a number of granzymes (granzyme A, B and K) (Fig. 3c,d). The upregulation of *IL-10* at day 69, but not day 7 was unexpected as upregulation of this cytokine has previously been reported to be universal in TpM, irrespective of lymphocyte lineage, and had been proposed to be associated with the dysregulation of the primary T-cell response discussed above^39^. However, our data suggests that the kinetics of the induction of IL-10 is not consistent with this hypothesis, as IL-10 expression is upregulated in attenuated but not pathogenic TpM. The upregulation of the granzyme genes in day 69 TpM cells is also of particular note as in a recent study by Rchiad et al.^14^, granzyme A (*GZMA*) was one of only 4 regulated genes consistently associated with an attenuated phenotype in the TaA. Additionally, the reduction in *GZMA* expression in the attenuated TaA was associated with reduced TGF-β signalling, which as commented above was also identified as a downregulated pathway at day 69 but not day 7 TpM, when compared to naïve, uninfected CD4^+^ T-cells. This adds further support to at least a component of the attenuation mechanisms being shared between *T. parva* and *T. annulata* transformed cells.

**Figure 4:**
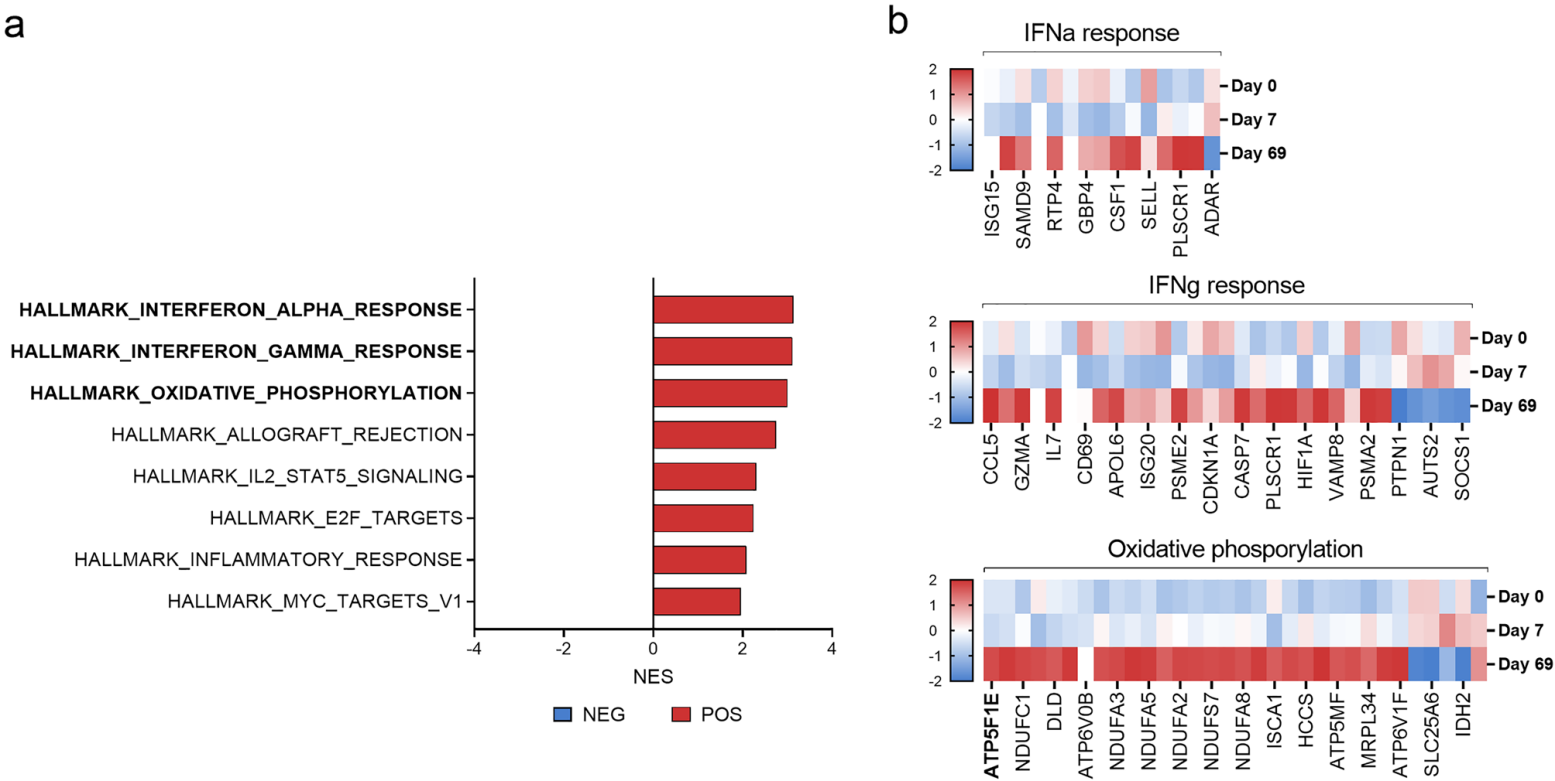
Gene set enrichment analysis of differentially expressed genes in attenuated vs pathogenic cells. (**a**) Normalized enrichment scores of gene sets that were either positively (red) or negatively (blue) significantly enriched at day 69 compared to day 7. (**b**) Expression of genes in selected significantly enriched gene sets day 69 compared to day 7. Expression is shown in z-scores of variance transformed counts. Created with Graphpad Prism v.9.3.1.

Functional annotation enrichment analysis and clustering using GSEA corroborated the positive enrichment of several gene sets related to immune processes (interferon-α (IFNA) response, IFNG response, allograft rejection, IL-2 STAT5 signalling and inflammatory response) in day 69 TpM when considering the global transcriptome (Fig. 4 and Supplementary Fig. 3). The only other enriched gene sets were oxidative phosphorylation (metabolism) and cell proliferation pathways (E2F targets and MYC targets V1). Metheni et al.^54^, proposed that attenuated TaA transition from the characteristic Warburg glycolysis to oxidative phosphorylation as cell proliferation slows, which our data suggests may also be happening in TpM (Fig. 4 and Supplementary File 2), drawing another parallel in the attenuation process between the two parasites. In their study, however, HIF1A was found to be up-regulated in both pathogenic and attenuated TaA as compared to negative controls^55^, whereas we found HIF1A only to be up-regulated when directly comparing attenuated to pathogenic cells. The enrichment of E2F targets and MYC targets V1 in the GSEA appeared counter-intuitive as, based on previous work by Morrison et al.^56^, and informal observations during general establishment and culture of TpM (unpublished), attenuated TpM were anticipated to have a reduced, rather than enhanced, proliferative phenotype. As GSEA doesn’t capture the directionality or interaction of genes, functional annotation clustering using DAVID was used to further explore and confirm the observations made with GSEA. The analysis at day 69 confirmed the upregulation of functional clusters including oxidative phosphorylation (and metabolic pathways) and immune functions (such as NK cell mediated cytotoxicity and chemokine signalling) but down-regulation of clusters that include cancer-like phenotypes and VEGF, ErbB, HIF-1 and Ras signalling pathways, which are closely associated with cell growth and proliferation^57–60^ (Table 2 and Supplementary File 3). Notably, an additional functional cluster found to be down-regulated at day 69 in this analysis included the PD-1 checkpoint pathway, a crucial immune checkpoint in cancer^61^, suggesting that attenuation of this pathway may also be associated with a decreased capacity of attenuated TpMs to inhibit, and thus evade elimination by T-cells.

**Table 2:** Summarized functional annotation clustering results.

Cluster	Direction	Score	Majority of terms related to (number/total)
1	Down	2.95	Cancers/cell proliferation (7/9)
2	Down	2.07	T-cell differentiation / regulation (2/3)
3	Down	1.98	Cancers/cell proliferation (21/71)
			Signalling (pathways) (19/71)
4	Down	1.75	Endocrinological processes (11/15)
5	Down	1.72	Cancers/cell proliferation (2/3)
1	Up	6.60	Cell metabolism (3/13)
			Degenerative diseases (6/13)
2	Up	2.29	(Auto-) Immunity (5/6)
3	Up	2.02	Signalling (pathways) (3/3)

This table includes only those clusters that were significantly enriched based on their enrichment score (the geometric mean of the negative log-adjusted *p*-values for each term in the cluster). An enrichment score of ≥ 1.3 was considered significant. The final column provides insight into the type of process the enriched terms in each cluster relate to most. See Supplementary File 3 for complete results. (https://david.ncifcrf.gov/; accessed on 25 April 2023).

**Figure 5:**
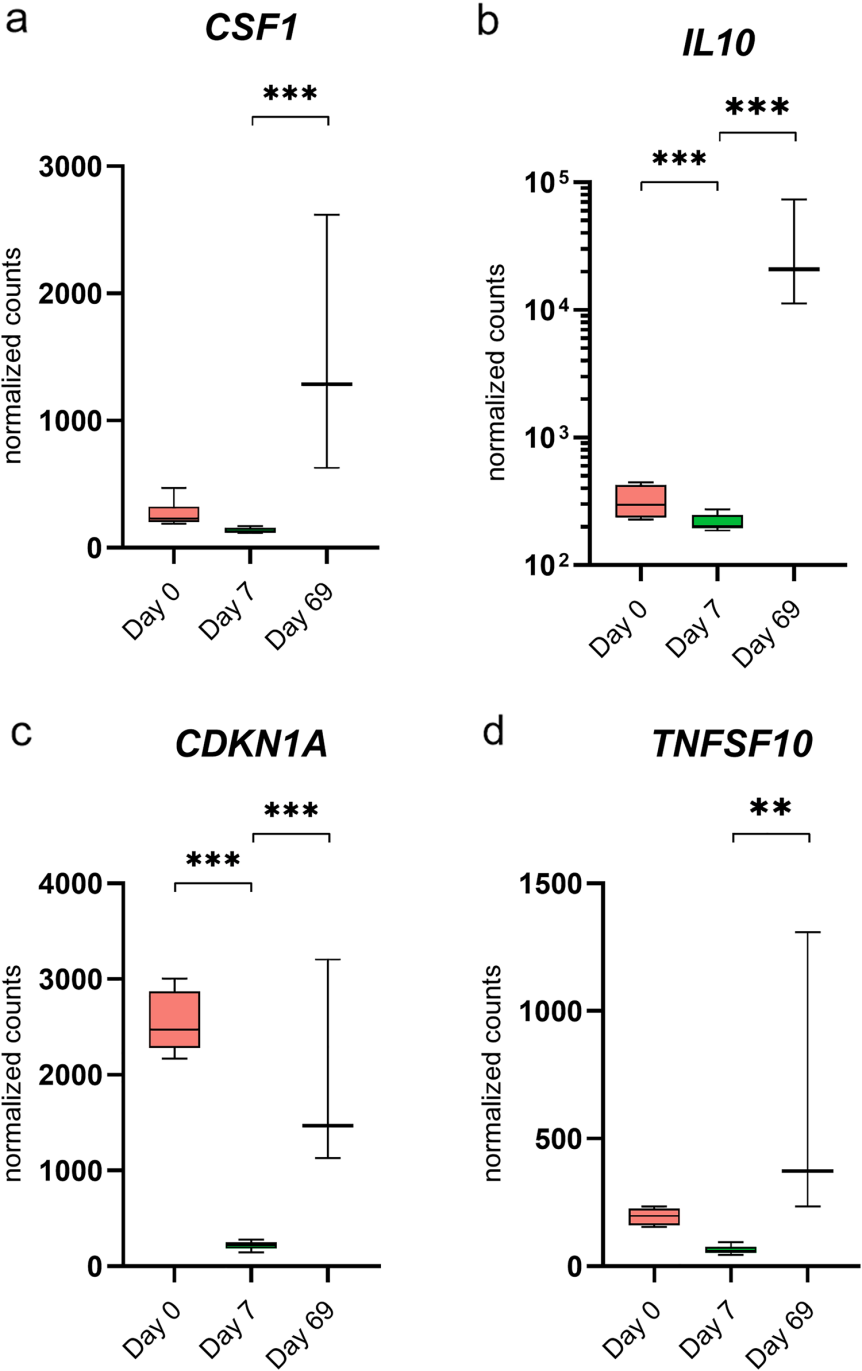
Expression of specific host genes identified in leading edge analysis. Box and whisker plots depicting the min–max values of normalized counts of *Bos taurus* genes (**a**) *CSF1*; (**b**) *IL10*; (**c**) *CDKN1A*; and (**d**) *TNFSF10*; expressed at day 0, day 7 and day 69. **p-value ≤ 0.01; ***p-value ≤ 0.001. Normalized counts of *IL10* are depicted on a log-scale Y-axis. Created with Graphpad Prism v.9.3.1.

Leading edge analysis was carried out to determine the key genes that may be driving attenuation. A number of genes formed part of the core enrichment of at least 3 of the aforementioned enriched gene sets (Table 3, Fig. 5 and Supplementary File 4). Of note, colony stimulating factor 1 (*CSF1*) emerged as the gene that contributed to the enrichment of the highest number of gene sets (allograft rejection, IFN-α response, inflammatory response and IL-2/STAT5 signalling). *CSF1* drives monocytes to produce a plethora of cytokines and as such plays a key role in innate immunity and inflammation^62^, suggesting CSF1 has a role in promoting the pro-inflammatory profile of attenuated TpM (Fig. 5a). However, previous work has shown GM-CSF (closely related to CSF1) to be expressed in TpM and part of an autocrine loop that can contribute to TpM proliferation^63^; such evidence points to the potentially contradictory roles that families of pleiotropic cytokines may play in determining the phenotypes of TpM. Similarly, interpretation of how IL10, a cytokine with multiple functions may influence TpM attenuation is complex due to evidence that in addition to its well-known anti-inflammatory roles, it can potentiate anti-cancer CD8^+^ T-cell responses^64–66^ and so may either enhance and/or diminish the immune response against day 69 TpM and contribute to or mitigate the attenuation phenotype (Fig. 5b). Cyclin-dependent kinase inhibitor 1A (*CKDN1A—*also known as p21), was among the leading edge of 5 enriched gene sets in our data. p21 is a known regulator of cell cycle progression, and has previously been described as having tumour suppressive properties, inducing anti-proliferative signals and senescense^67^ (Fig. 5c). Therefore, upregulation of *CDKN1A* in attenuated cells may contribute to a mitigation of the proliferative state. However, similar to *CSF1* and *IL-10*, p21 has also been reported to have contradictory effects, with proto-oncogenic activity also having been observed^67^. The TNF Superfamily Member 10 (*TNFSF10*—also known as tumour necrosis factor-related apoptosis-inducing ligand (TRAIL)), which was enriched in 4 of the top gene sets, has been shown to induce apoptotic cell death in cancerous and transformed cells^68^ and so, unlike the other genes discussed here, there is an unambiguous potential to contribute to the attenuation of TpM at day 69 (Fig. 5d).

**Table 3:** Leading edge analysis of the significantly enriched gene sets.

Symbol	Description	Function	Number of gene sets	Gene sets (Hallmark)
*CSF1*	Colony stimulating factor 1	Regulation of survival, proliferation and differentiation of hematopoietic precursor cells and release of pro-inflammatory chemokines	4	Ar, IαR, Ir, ISs
*CKDN1A*	Cyclin dependent kinase inhibitor 1A	A regulator of cell cycle progression at G1	3	E, IγR, Ir
*TNFSF10*	TNF Superfamily Member 10	Induces apoptosis in transformed and tumor cells	3	IγR, Ir, ISs
*BST2*	Bone Marrow Stromal Cell Antigen 2	*Undetermined*	3	IαR, IγR, Ir
*SELL*	Selectin L	A cell surface adhesion molecule	3	IαR, Ir, ISs
*IL7*	Interleukin 7	A cytokine important for B- and T-cell development	3	Ar, IγR, IαR
*RTP4*	Receptor Transporter Protein 4	Involved in defense response to virus	3	IαR, IγR, Ir
*IL10*	Interleukin 10	Cytokine with pleiotropic effects in immunoregulation and inflammation	3	Ar, Ir, ISs
*GBP4*	Guanylate Binding Protein 4	Induced by interferon, hydrolyses GTP to GDP and GMP	3	IαR, IγR, ISs
*CCL5*	C–C Motif Chemokine Ligand 5	Chemoattractant for blood monocytes, T helper cells and eosinophils	3	Ar, IγR, Ir

Leading edge analysis was performed only on those gene sets that were significantly enriched as determined by the pre-ranked GSEA. Only genes that formed part of the leading edge in ≥ 3 gene sets are listed in this table. The order of genes is descending according to number of gene sets they enriched. Description and function of genes derived from GeneCards—the human gene database (https://www.genecards.org/)^69,70^. Gene sets = each gene set in which the gene formed part of the core enrichment. Gene sets: Ar = allograft rejection; E = E2F targets; IαR = IFNα response; IγR = IFNγ response; Ir = inflammatory response; ISs = IL2/STAT5 signalling.

### Prolonged in vitro culture induces alterations in *T*. *parva* gene expression

**Figure 6:**
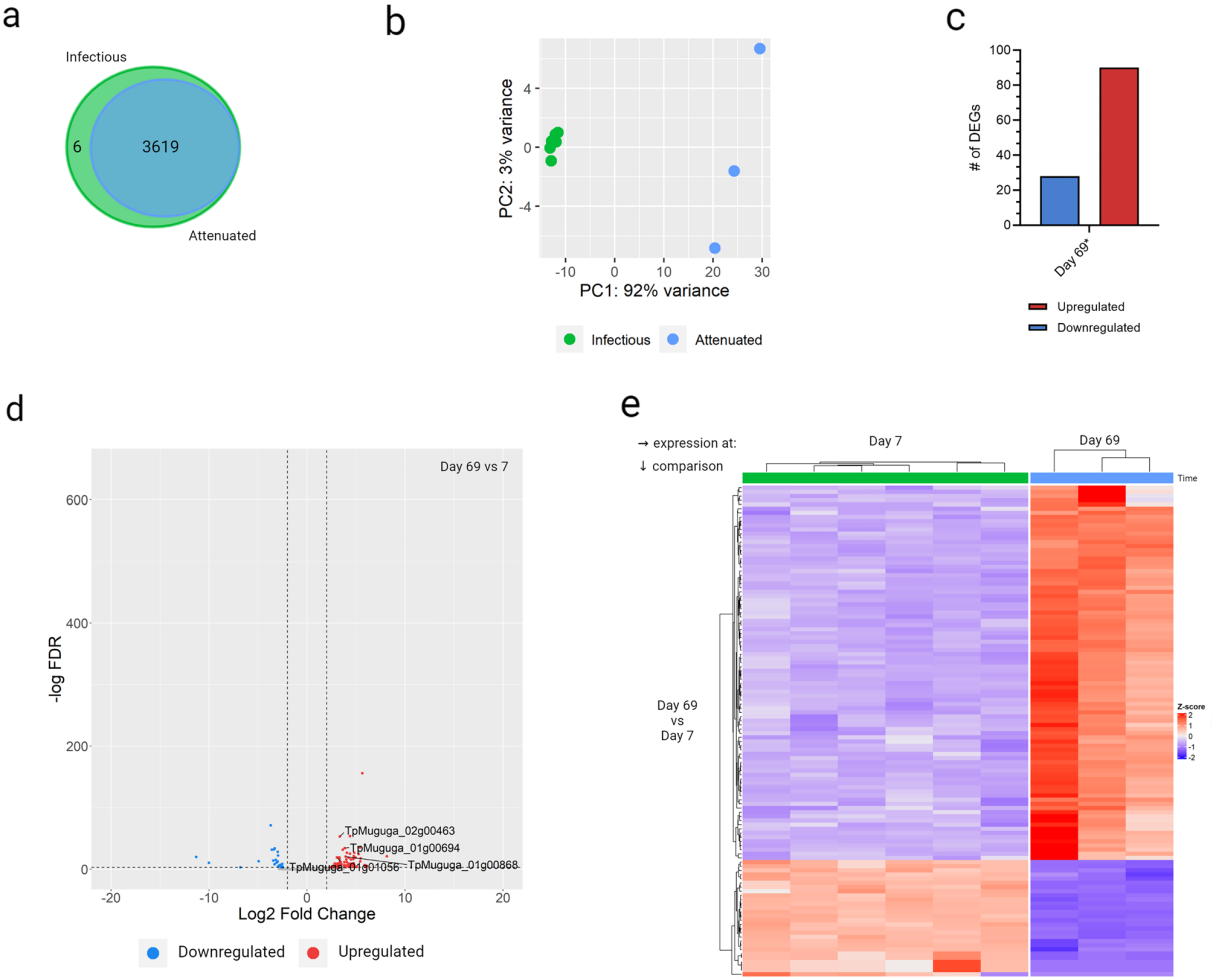
Expression of *Theileria parva* genes in different states of *T. parva* infection of bovine CD4^+^ T-cells. (**a**) The total number of expressed genes at day 7 (green) and day 69 (blue). (**b**) Principal component analysis plot showing the first and second principle components; based on variance stabilized transformed counts of all expressed genes. (**c**) The number of significantly upregulated (red) or downregulated (blue) genes day 69 compared to day 7, as determined by DE analysis using DESeq2 (FDR ≤ 0.05 and L2FC |≤ 2|). (**d**) Distribution of all significantly DEGs according to the L2FC and -log(adjusted p-value) at day 69 compared to day 7. (**e**) Expression of the all significantly DEGs at day 69 compared to day 7, displayed as z-score transformed counts. Created with ComplexHeatmap v2.12.1 and ggplot2 v3.4.2 in R Studio v2023.03.1, Graphpad Prism v9.3.1 and BioRender.com.

To explore if the transition to attenuation of the TpM was associated with changes in *T. parva* transcriptome*,* we compared the gene expression patterns observed at day 7 and day 69. After quality control, an average of 1.4 (3.3%) and 3.1M (10.0%) reads uniquely aligned to the *T. parva* reference transcriptome in pathogenic (day 7) and attenuated (day 69) TpM, respectively. Of the 4129 defined *T. parva* genes^22^, 3625 genes (> 87%) were represented in the transcriptome dataset (with a minimum of 10 unique mapped reads per gene). Most of the expressed genes overlapped between day 7 and day 69, the only exception being 6 genes uniquely expressed in pathogenic cells (Fig. 6a and Supplementary File 5). As with analysis of the bovine genes, PC analysis of the *T. parva* transcriptomes revealed clustering of samples according to days post-infection (Fig. 6b). Of the 3625 genes analysed only 118 (3%) genes were significantly differentially expressed (FDR ≤ 0.05 and L2FC of ≥ 2). The majority (90/118) of DEGs were upregulated in attenuated cells, whilst only 28 DEGs were downregulated (Fig. 6c). A hierarchical clustering heatmap of all DEGs highlighted that the DEGs were remarkably consistent across all samples at the same time-point (Fig. 6e).

**Figure 7:**
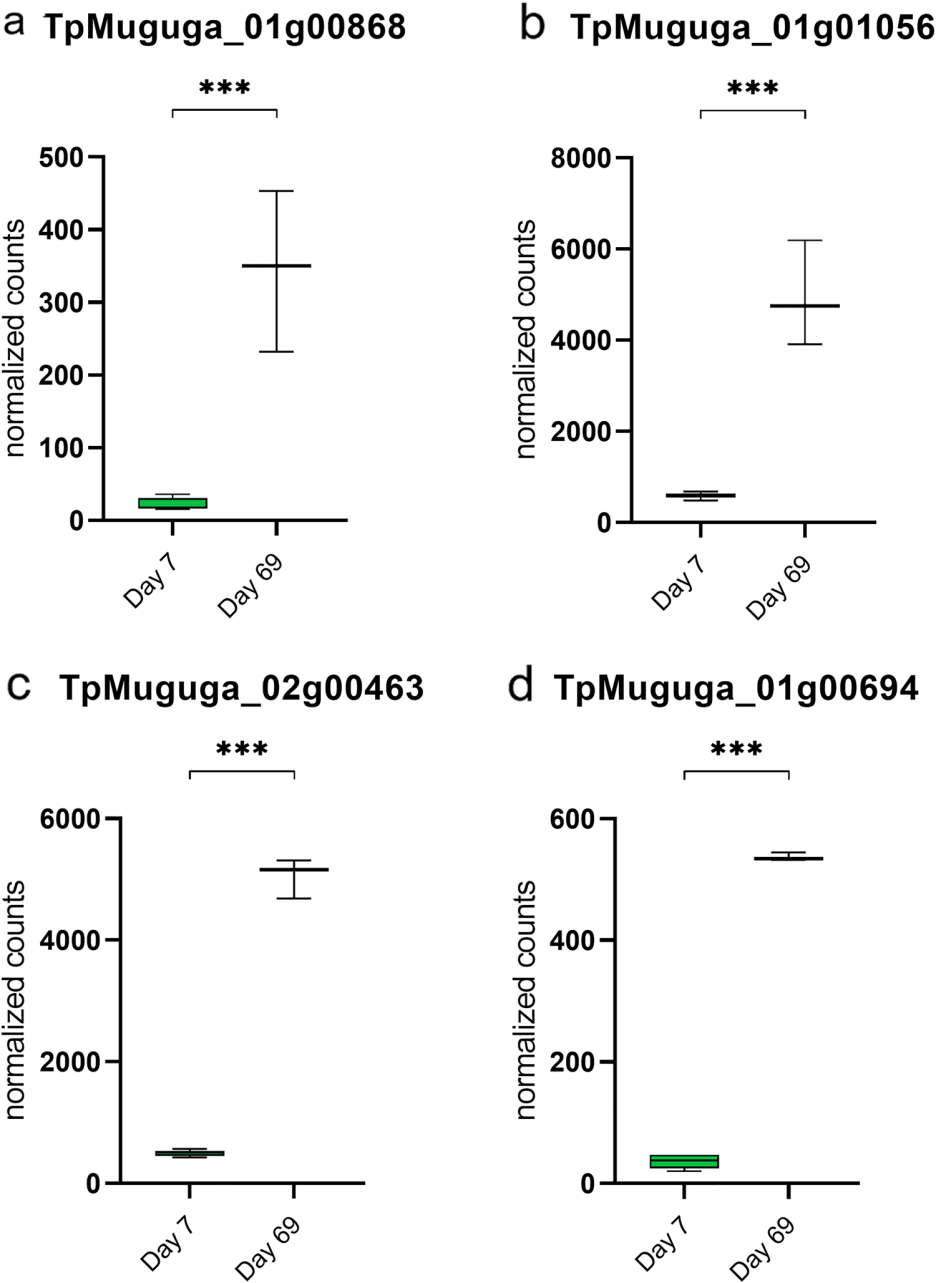
Expression of specific parasite genes in significantly enriched pathways or gene sets in attenuated cells. Box and whisker plots depicting the min–max values of normalized counts of *Theileria parva* genes (**a**) TpMuguga_01g00868; (**b**) TpMuguga_01g01056; (**c**) TpMuguga_02g00463; and (**d**) TpMuguga_01g00694; expressed at day 7 and day 69. ***p-value ≤ 0.001. Created with Graphpad Prism v.9.3.1.

Most of the characterised *T. parva* antigens (including Tp1-10, Tp12-36, p32 and p67)^71–73^ were non-differentially expressed at day 7 and day 69—the only exceptions were Tp3 (TpMuguga_01g00868) and p32 (TpMuguga_01g01056), which were both upregulated at day 69 (Figs. 6d, 7a and b, and Supplementary File 6). The upregulation of p32, which is a merozoite surface antigen^74^, at day 69 fits in with the biological life-cycle of the parasite (i.e. upregulation of a merozoite antigen would be anticipated to occur late, rather than early, in the schizont phase), as does the absence of p67 expression at either time-point, which has previously been considered a sporozoites-specific antigen^75^. New evidence by Tonui, et al.^76^ is now challenging this view, who did find low levels of expression of p67 in the schizont, which is in conflict with our findings. The finding that most antigens were expressed at equivalent levels at day 7 and day 69 suggests that differential expression of antigens (and so potential exposure to *T. parva*-specific B-cell or T-cell responses) was not evidently a mechanism contributing to attenuation of the TpM.

Of the 118 differentially regulated genes there were two major protein groupings. The first of these were genes encoding ribosomal proteins (n = 9), all of which were downregulated at day 69; notably 3/6 of the *T. parva* genes that were expressed at day 7 but not at day 69 also encoded ribosomal proteins (Supplementary File 5). Therefore, ribosomal downregulation, probably reflecting a relative decrease in parasite protein production in attenuated cells, was a general feature of day 69 cells. The second group was membrane proteins (n = 22, inner membrane complex proteins/(putative) integral membrane proteins) of which all but one were upregulated at day 69; this probably reflects the increase in the size of the schizonts in infected cells later in the infection, the consequent increase in the surface area of the schizonts, and so a need for greater quantities of membrane proteins. It is likely that the relative downregulation of ribosomes and upregulation of membrane proteins seen at day 69 are consequences of attenuation rather than changes that drive the attenuation phenotype.

Of the remaining 87 differentially expressed genes only approximately half (n = 43) encoded annotated proteins, none of which could be easily linked to functional activities that may be related to attenuation. However, upregulation of *RCD1* (TpMuguga_02g00463) and a histidine protein (TpMuguga_01g00694) (Figs. 6d, 7c and d), both of which are involved in the control of reactive oxygen species (ROS) in plants^77,78^, may be linked to the oxidative stress and subsequent altered metabolic state of the cells as observed in the host transcriptome. Due to the small number of annotated differentially expressed *T. parva* genes, functional analysis using the Gene Ontology Enrichment tool on PiroplasmaDB (VeuPathDB^21^) did not provide additional insight (data not shown). The remaining 44 differentially regulated ‘hypothetical proteins’ (38 upregulated and 6 downregulated at day 69), for which there is obviously no implied functional information available, may therefore be of particular interest, as it is feasible to suppose that the proteins contributing to attenuation may be encoded by genes within this set of uncharacterised genes. In order to further investigate possible functions of these proteins, the search tool on PiroplasmDB (VeuPathDB^21^) was used to identify possible orthologs in *T. annulata*. Only 9 of the unannotated *T. parva* genes were annotated in *T. annulata*, but their functions did not provide additional clarity on their relevance in the present study (Supplementary File 7). To provide clarity on this, additional work applying novel approaches to provide some characterisation of the structure, orthology and/or function of these genes, would be required^79,80^.

### Limitations of the study

At the commencement of this study there was no transcriptional data profiling attenuated vs pathogenic *T. parva*-infected cells, and the rationale of this study was thus to fill this knowledge gap. Due to the fragility of early passage *T. parva* cells, meaning they can’t be reliably cryopreserved, it was not possible to retrospectively perform phenotypic analyses, based on the transcriptome data, of the cells at day 7. However, the data from this study provides a strong platform to select and integrate relevant phenotypic assays in future iterations of the experiments, which are currently being planned. Thus, whilst phenotypic confirmation of the traits implied by the transcriptional profiles was not possible within this study, they will form core components of future work.

As a consequence of the SARS-CoV-2 pandemic the animals involved in this study were culled prior to day 69 of the experiment and so it was not possible to administer the autologous cell-lines in vivo at the day 69 timepoint. Although this precluded our ability to empirically confirm that the cells were attenuated at this time, we are confident that the cells would have had an attenuated phenotype. This is based on additional information from Prof. W.I. Morrison that *T. parva*-infected cell-lines are reliably attenuated within 42 days of passage (personal communication—which extends the observations made in published data^9^) and our own experience where in vitro established cell-lines are used as a means of immunizing animals without the need for concurrent treatment. We are aware that this timeline is distinct from that observed in *T. annulata*, where a longer period of in vitro culture is required to guarantee attenuation.

## Conclusion

Our data indicates that attenuation of *T. parva*-infected cells is associated with a decrease in proliferation and a restoration of an inflammatory profile, as well as a change in the metabolism of TpM. These transcriptomic changes broadly fit with observations made on the phenotype of TpM and can be rationalised as contributing to attenuation (i.e. it is intuitive that a reduction in proliferation and a mitigation of the anti-inflammatory profile may facilitate attenuation). Perhaps surprisingly, attenuation was associated with an increase in the perturbation of the transcriptomic landscape of the host cell, with many more genes differentially regulated at day 69 than at day 7, suggesting attenuation is a progressive process with a greater divergence from, rather than a partial reversion to, the transcriptome of uninfected T-cells.

Some of the differentially regulated host genes identified in this study (e.g. *TRAIL*, *PD-1*, *TGF-β*, granzymes), are particularly attractive candidates for further exploration, as they have also been identified in attenuated *T. annulata*-infected cell-lines. The finding that only a small fraction of the *T. parva* genes were differentially regulated between the pathogenic and attenuated TpM is encouraging and provides a restricted number of genes that are candidates for further exploration. Which of these host and pathogen genes (or pathways) drive attenuation requires further functional and transcriptomic studies to provide better resolution. Fortunately, the *T. parva* model provides a number of opportunities for further comparative transcriptomic analyses. For example comparison of B-cell TpM (which are effectively attenuated very early after in vitro infection) with T-cell TpM (and even T-cell subsets). The fact that both *T. parva* and *T. annulata* can infect B-cells offers opportunities for direct cross-species comparisons; notably, some of the transcriptomic changes seen in attenuated TpM are similar to those observed in TaA (e.g. granzyme upregulation in attenuated cells), whilst others appear to diverge between the species. Thus, although transcriptional changes cannot be extrapolated across these two parasites (*T. parva* and *T. annulata*) a better understanding of the commonalities and differences could provide novel molecular insights into the attenuation process. Comparison of transcriptomic differences of *T. parva*-infected cells derived from animals of the recently identified *T. parva* tolerant family lineage^81^ from those derived from susceptible animals may be interesting to see if tolerance is mediated through similar gene regulation as that observed with attenuation (notably tolerance has been associated with a reduced proliferative capacity of TpM^82^). Combining greater understanding of transcriptomic changes associated with attenuation and novel approaches to genetic manipulation will offer new insights on the host/pathogen interaction and lead to new methods that can be exploited to control these important livestock pathogens.

### Supplementary Information

Supplementary Figure 1.

Supplementary Figure 2.

Supplementary Figure 3.

Supplementary Information 1.

Supplementary Information 2.

Supplementary Information 3.

Supplementary Information 4.

Supplementary Information 5.

Supplementary Information 6.

Supplementary Information 7.

Supplementary Legends.

## Data Availability

The raw and processed data are available through GEO accession number GSE245180.
