# Supplementary figures and images for: Comparative transcriptional analysis identifies genes associated with the attenuation of Theileria parva infected cells after long-term in vitro culture
Source: Sci Rep. 2024 Apr 18;14:8976. doi: 10.1038/s41598-024-59197-y (PMC11026401; doi:10.1038/s41598-024-59197-y)

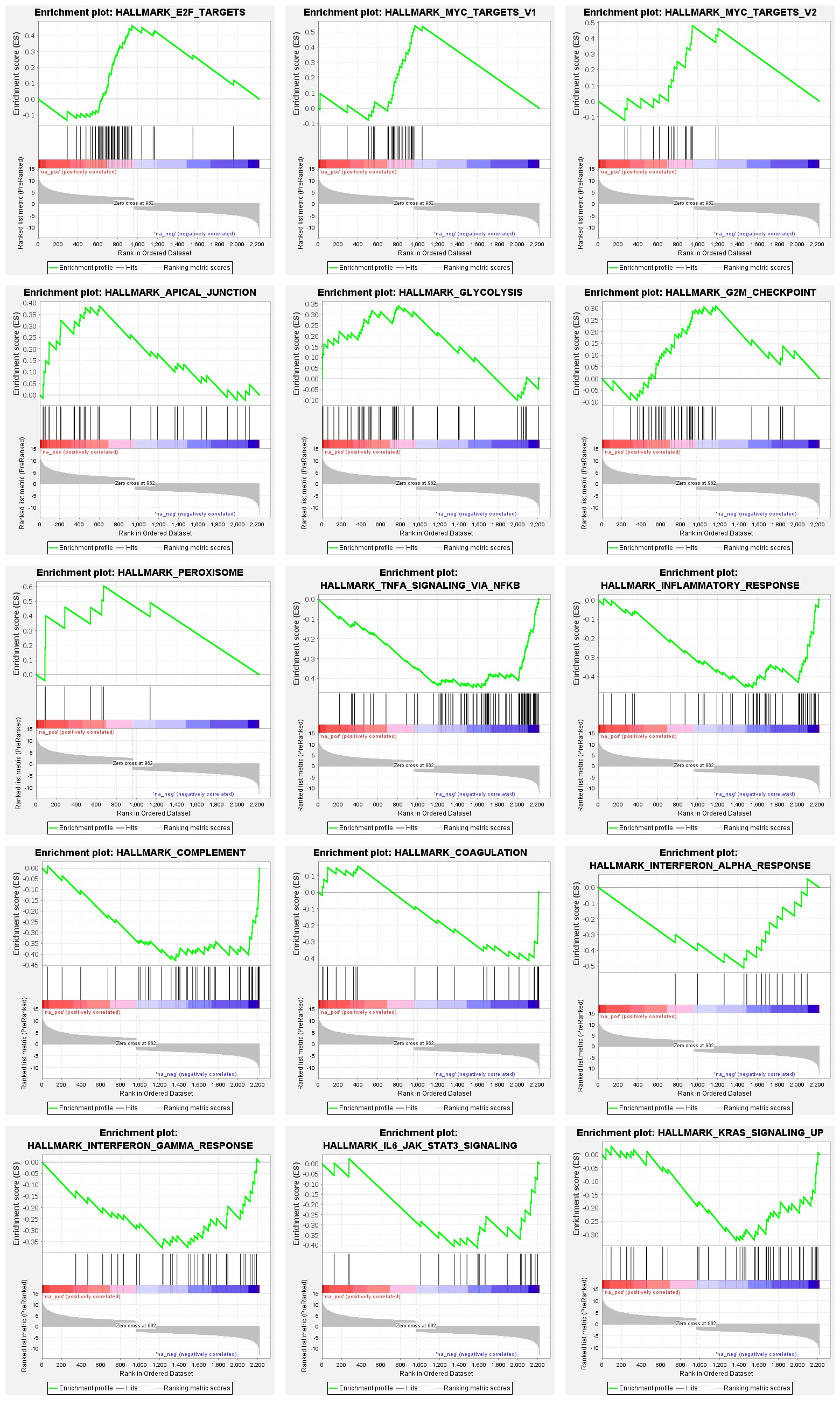

Supplement: Supplementary file 1 — Supplementary Figure 1. [file 41598_2024_59197_MOESM1_ESM.tif]

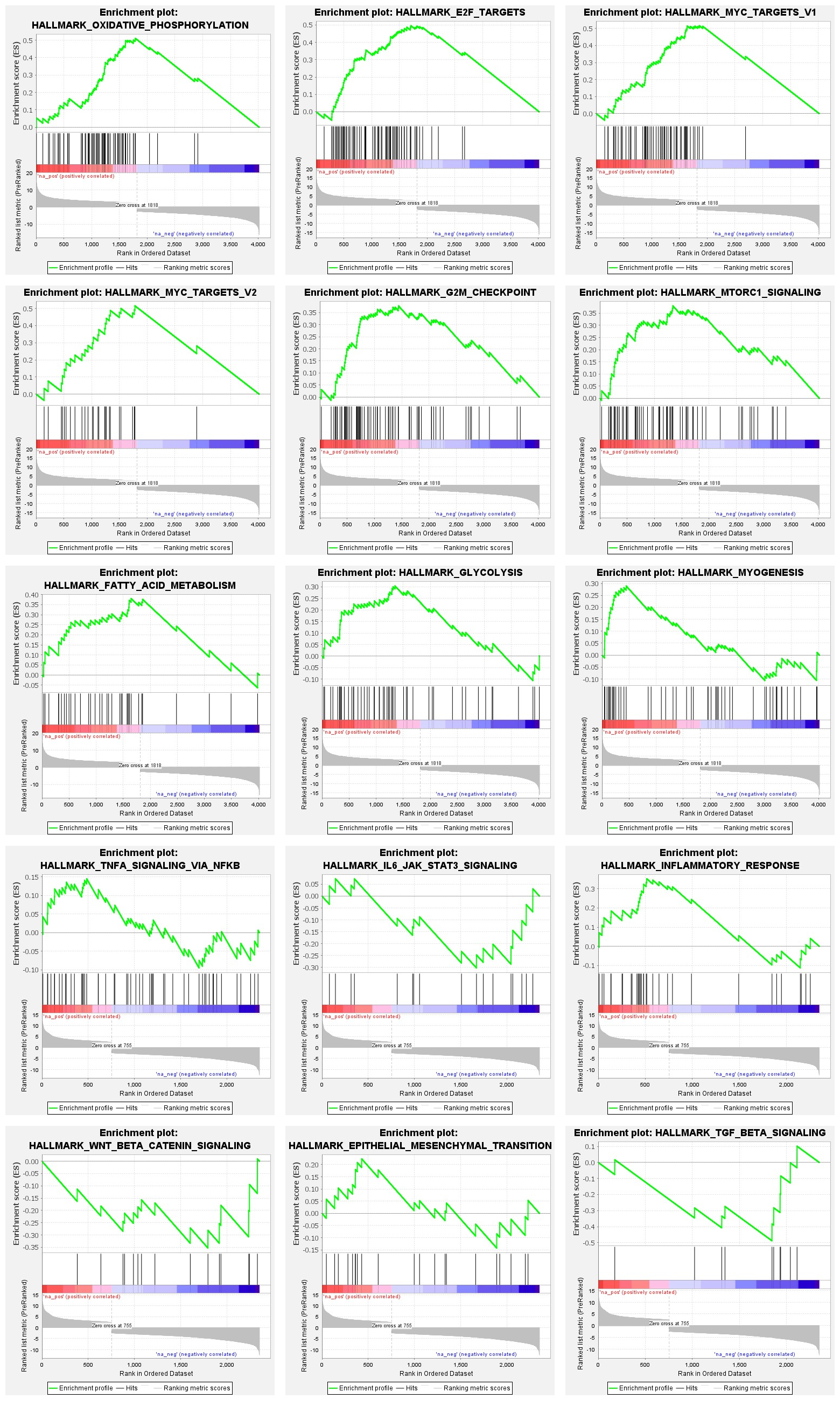

Supplement: Supplementary file 2 — Supplementary Figure 2. [file 41598_2024_59197_MOESM2_ESM.tif]

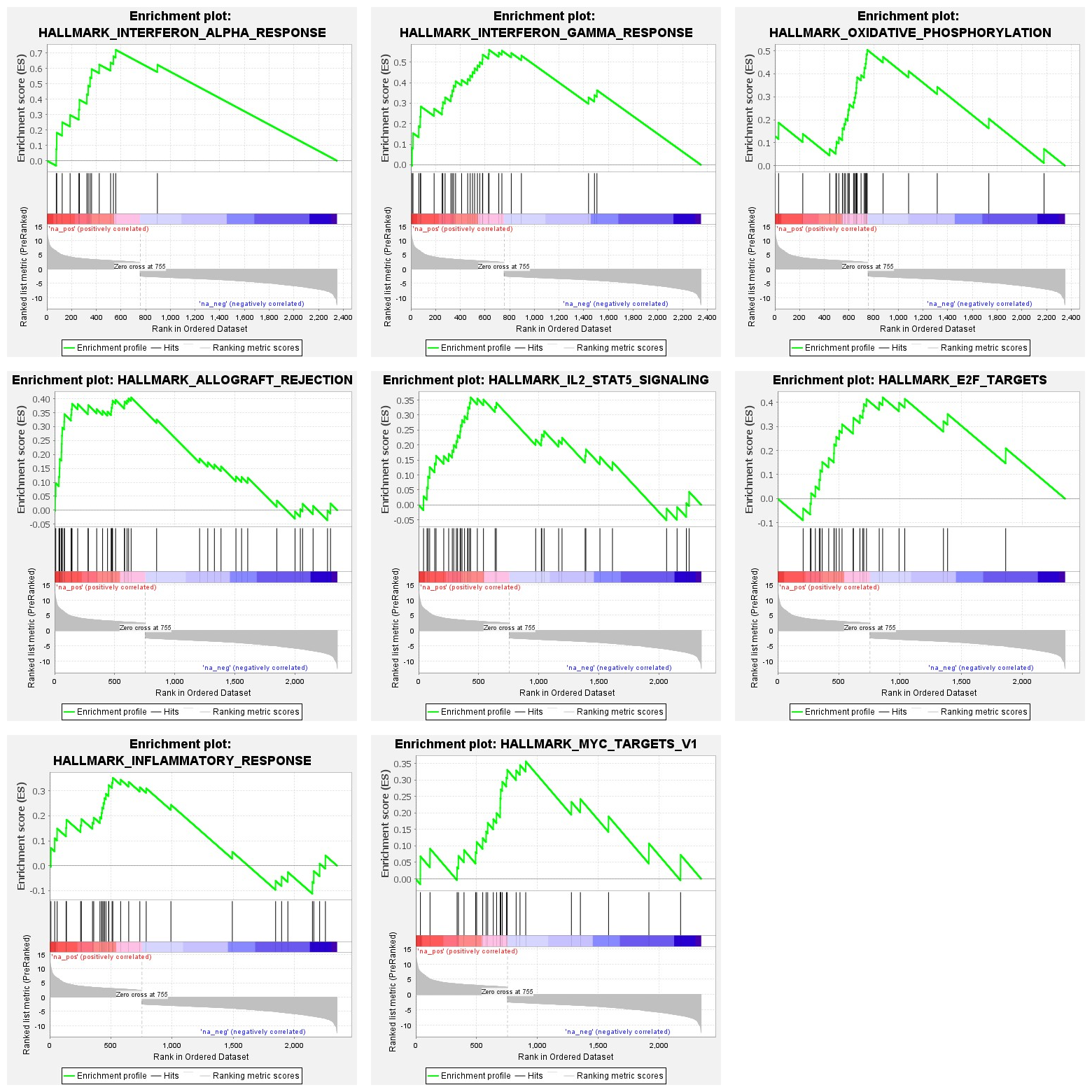

Supplement: Supplementary file 3 — Supplementary Figure 3. [file 41598_2024_59197_MOESM3_ESM.tif]
