# Supplementary material for: Comparative transcriptional analysis identifies genes associated with the attenuation of Theileria parva infected cells after long-term in vitro culture
Source: Sci Rep. 2024 Apr 18;14:8976. doi: 10.1038/s41598-024-59197-y (PMC11026401; doi:10.1038/s41598-024-59197-y)
Supplement: Supplementary file 11 — Supplementary Legends. [file 41598_2024_59197_MOESM11_ESM.docx]

# Supplementary files and figures

**Supplementary File 1. Outcome of differential expression analysis.** The expression of host (*Bos taurus*) and parasite (*Theileria parva*) genes was analysed between the different timepoints of interest using DESeq2 in R Studio. Bos_taurus_DEGs = all *Bos taurus* genes that were expressed at day 7 or day 69, relative to day 0 (termed day7vs0 and day69vs0 respectively), or at day 69, relative to day 7 (termed day69vs7). Theileria_parva_DEGs = all *Theileria parva* genes that were expressed at day 69, relative to day 7. Genes were considered differentially expressed at log2 fold change (L2FC) ≥2 and false discovery rate (FDR) ≤0.05. For each gene the Gene ID, Symbol (*B. taurus* only) and description is provided along with the log 2 fold change (L2FC) and false discovery rate (FDR) of the relevant comparison, frequency (number of comparisons in which the gene was found to be differentially expressed), and the normalized counts in each sample. Gene symbols and descriptions were derived from the Ensembl biomart database^19^.

**Supplementary File 2.** **Gene set enrichment analysis outcomes.** Gene sets that were significantly enriched by the list of DESEq2 pre-ranked DEGs. Gene sets analysed were the hallmark gene sets^34^ from the Molecular Signature Database^35^. Only gene sets with FDR-values of ≤ 0.05 were considered significantly enriched. Day7vs0 = day 7 relative to day 0; Day69vs0 = day 69 relative to day 0; Day 69vs7 = day 69 relative to day 7. Size = number of expressed genes in gene set; ES = enrichment score; NS = normalized enrichment score; FDR = false discovery rate.

**Supplementary File 3. Functional annotation clustering analysis results.** Clusters that were significantly enriched based on their enrichment score (the geometric mean of the negative log-adjusted *p*-values for each term in the cluster). Therefore, an enrichment score of ≥1.3 was considered significant. The reported *p*-values and Benjamini Hochberg (BH)-adjusted *p*-values are derived from the original functional annotation tool outcomes within the DAVID suite. Enrichment score = overall enrichment score for the cluster; Pathway map identifier = KEGG^29-31^ identifier; Count = number of DEGs in our dataset present in the pathway; Genes = Gene_IDs of DEGs in our dataset present in the pathway; Fold enrichment = fold enrichment of the individual pathways within the cluster. (<https://david.ncifcrf.gov/>; accessed on 25 April 2023).

**Supplementary File 4. Leading edge analysis of the significantly enriched gene sets in attenuated cells.** Overview of the gene sets and signals used in the leading edge analysis. Generated using GSEA-P (v 4.3.2)^33^.

**Supplementary File 5. Uniquely expressed and top differentially expressed *Theileria parva* genes.** Uniquely_expressed_genes = those genes that were uniquely expressed in pathogenic cells (day 7). The gene name and description are provided. Top_DEGs = the top 20 up- and top 20 down-regulated DEGs. Terms for gene ontology (GO) cell component (CC), biological process (BP) and molecular function (MF) is provided where known^21^. The log 2 fold change (L2FC) and false discovery rate (FDR) are provided.

**Supplementary File 6. Expression of known *Theileria parva* antigens.** Overview of the expression of several well-described *T. parva* antigens at day 7 and day 69.

**Supplementary File 7. Theileria annulata orthologs of differentially expressed T. parva genes.** Overview of the orthologs in *T. annulata* of the 118 *T. parva* DEGs between day 69 vs day 7 based on PiroplasmDB (VeuPathDB^21^).

**Supplementary Figure 1. Pre-ranked Gene Set Enrichment Analysis of significant DEGs in pathogenic cells.** Enrichment plots of gene sets that were significantly positively enriched in pathogenic cells (day 7), when compared to uninfected cells (day 0). The top panel of each plot displays the running enrichment score (ES) for the gene set as the analysis walks down the ranked list. The final ES for the gene set is the peak of this plot. The middle panel depicts the location of members of the gene set in the pre-ranked list of genes. The bottom panel indicates the value of the ranking metric as you move down the list of pre-ranked genes. The normalized ESs for each hallmark gene set are listed in Supplementary File 2.

**Supplementary Figure 2. Pre-ranked Gene Set Enrichment Analysis of significant DEGs in attenuated cells.** Enrichment plots of all gene sets that were significantly enriched in attenuated cells (day 69), when compared to uninfected cells (day 0). The top panel of each plot displays the running enrichment score (ES) for the gene set as the analysis walks down the ranked list. The final ES for the gene set is the peak of this plot. The middle panel depicts the location of members of the gene set in the pre-ranked list of genes. The bottom panel indicates the value of the ranking metric as you move down the list of pre-ranked genes. The normalized ESs for each hallmark gene set are listed in Supplementary File 2.

**Supplementary Figure 3. Pre-ranked Gene Set Enrichment Analysis of significant DEGs in attenuated cells.** Enrichment plots of all gene sets that were significantly enriched in attenuated cells (day 69), when compared to pathogenic cells (day 7). The top panel of each plot displays the running enrichment score (ES) for the gene set as the analysis walks down the ranked list. The final ES for the gene set is the peak of this plot. The middle panel depicts the location of members of the gene set in the pre-ranked list of genes. The bottom panel indicates the value of the ranking metric as you move down the list of pre-ranked genes. The normalized ESs for each hallmark gene set are listed in Supplementary File 2.
